# Natural genetic variation in a dopamine receptor is associated with variation in female fertility in *Drosophila melanogaster*

**DOI:** 10.1098/rspb.2023.0375

**Published:** 2023-04-12

**Authors:** Richard F. Lyman, Rachel A. Lyman, Akihiko Yamamoto, Wen Huang, Susan T. Harbison, Shanshan Zhou, Robert R. H. Anholt, Trudy F. C. Mackay

**Affiliations:** Program in Genetics, W. M. Keck Center for Behavioral Biology and Department of Biological Sciences, North Carolina State University, Raleigh, NC 27695, USA

**Keywords:** diallel cross design, GWAS, RNAi, *Dop2R*

## Abstract

Fertility is a major component of fitness but its genetic architecture remains poorly understood. Using a full diallel cross of 50 *Drosophila* Genetic Reference Panel inbred lines with whole genome sequences, we found substantial genetic variation in fertility largely attributable to females. We mapped genes associated with variation in female fertility by genome-wide association analysis of common variants in the fly genome. Validation of candidate genes by RNAi knockdown confirmed the role of the dopamine 2-like receptor (*Dop2R*) in promoting egg laying. We replicated the *Dop2R* effect in an independently collected productivity dataset and showed that the effect of the *Dop2R* variant was mediated in part by regulatory gene expression variation. This study demonstrates the strong potential of genome-wide association analysis in this diverse panel of inbred strains and subsequent functional analyses for understanding the genetic architecture of fitness traits.

## 1. Introduction

Additive genetic variation for fitness determines the response of populations to natural selection [1,2]. The response of a fitness component to natural selection depends on its additive covariance with fitness, which in turn depends on its heritability and genetic correlation with fitness [2,3]. Understanding the magnitude of genetic variation for fitness and its components and the mechanisms maintaining this genetic variation is a major goal of evolutionary quantitative genetics [4,5]. Individual fitness is defined as the contribution of offspring to the next generation and is determined by the individual's viability (survival past reproductive age) and fertility (number of offspring produced). Estimates of genetic variation for viability and fertility in wild populations are confounded by potential genotype by environment correlation and genotype by environment interaction, and the inability to separate male and female effects on fitness. Therefore, much of our empirical knowledge about the genetic basis of variation for fitness and its components is based on laboratory studies, in particular on *Drosophila melanogaster* [6]. The fertility component of fitness is genetically variable in *D. melanogaster* [7–12]; however, the genetic architecture of fertility and the specific genes and variants that contribute to genetic variation in fertility remain poorly understood. Here, we characterize the genetic basis of variation in fertility of young *D. melanogaster* from the sequenced, inbred lines of the *Drosophila* Genetic Reference Panel (DGRP) [13,14] using a combination of a classical diallel cross design to estimate variance components, high-resolution association mapping to identify candidate genes and variants, and RNA interference (RNAi) of candidate genes and variant-based functional validation. We find a previously undocumented role of a dopamine receptor affecting natural genetic variation in fertility.

## 2. Results and discussion

**Figure 1.**
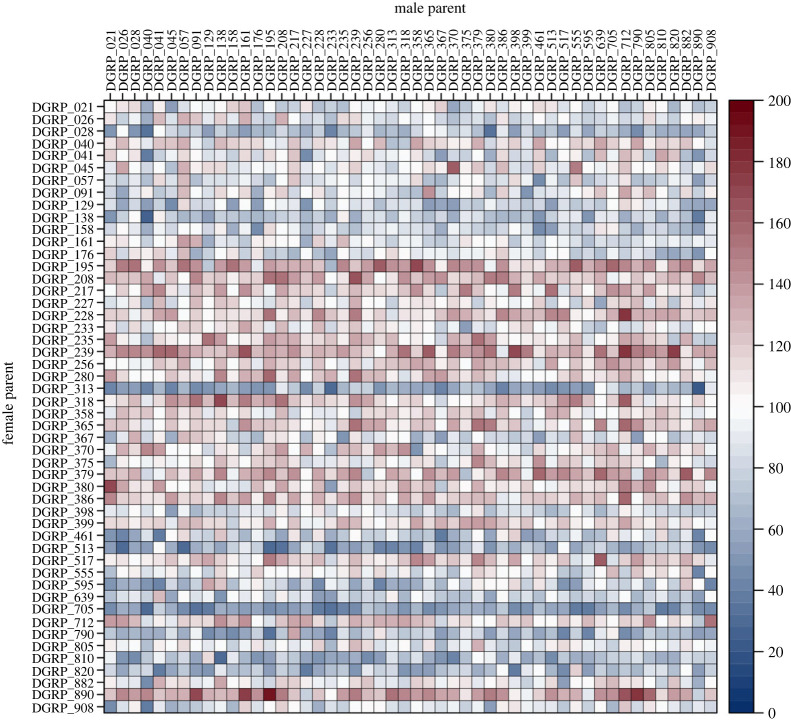
Variation in productivity among crosses of genetically diverse inbred lines. Each cell of the matrix represents one of the 2500 possible crosses of 50 DGRP inbred lines crossed in a full diallel mating design, with the lines used as sires and dams indicated on the edges of the matrix. The cells are colour-coded according to the mean productivity (total number of adult offspring) of three independent replicate crosses, as indicated by the heatmap of productivity.

We used a full diallel cross design (reciprocal crosses of parental lines with self-crosses included) of 50 DGRP lines to estimate genetic and environmental variance components for fertility. We defined a quantitative trait closely related to fertility of young flies—productivity—as the number of males and females emerging from crosses of four females and four males of the parental lines, with egg laying restricted to 48 h to minimize larval competition. We measured productivity in three replicate vials for each of the 50 × 50 = 2500 possible crosses (figure 1), scoring 7 37 868 flies in total. We did not find evidence of sex ratio bias (electronic supplementary material, figure S1) and therefore analysed the total number of female and male progeny in the 7500 replicate vials. Approximately 50% of the DGRP lines are infected with the symbiont *Wolbachia pipientis* [14]. We tested the effect of *Wolbachia* infection on productivity and found a small but statistically significant reduction in productivity for crosses when either parent was infected (electronic supplementary material, table S1 and figure S2). Infected females have similar reductions in productivity regardless of the Wolbachia status of the male mate (a decrease of approx. six adults on average), while uninfected females have decreased productivity (approx. three adults on average) when mated to infected males compared to mating with uninfected males. Overall, Wolbachia infection affects productivity, but the effect is greater when the females are infected. This accounts for the large female parent by male parent infection status interaction (electronic supplementary material, table S1). Therefore, we adjusted productivity for the *Wolbachia* infection status of parents before further analyses.

**Figure 2.**
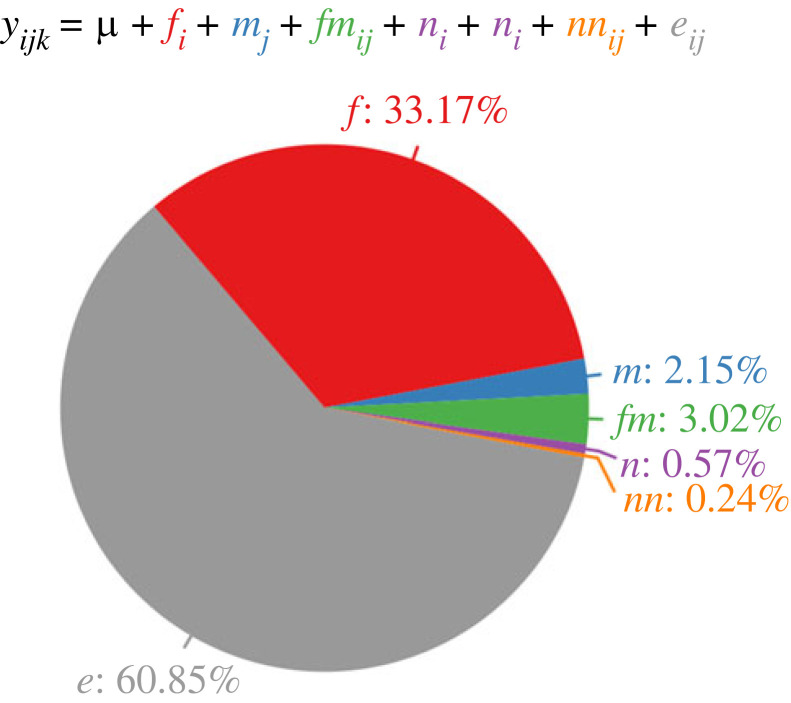
Sources of variation in productivity. The pie chart shows the relative contributions of variance components for productivity from the diallel cross. Variance components were estimated using the mixed effects model $y_{ijk}=\mu +f_{i}+m_{j}+fm_{ij}+n_{i}+n_{j}+nn_{ij}+\varepsilon_{ijk}$, where μ is the fixed effect for the grand mean; *f*, *m*, fm, *n*, nn and ε are random effects for the female extranuclear, male extranuclear, extranuclear interaction, nuclear, nuclear interaction and the residual effects, respectively (see text for explanation) and the subscript for *y* indexes the *i*th female, *j*th male and *k*th replicate.

We observed considerable phenotypic variation in productivity among the crosses. The distribution of productivity appeared symmetrical and normal with a coefficient of variation of 37% (electronic supplementary material, figure S3). Importantly, 39% of the total variation in productivity was attributable to non-environmental factors, i.e. parents of the crosses (electronic supplementary material, table S2). The fact that parents of the crosses differ only by their genotypes implies that the non-environmental variance component is entirely genetic. The genetic variation of productivity observed here includes genetic variation among the female and male parents as well as genetic variation among the progeny. Inbreeding of progeny (from self-crosses) appeared to have negligible effect (*p* = 0.08, electronic supplementary material, table S3). We further partitioned the genetic variation among crosses according to the bio-model of Cockerham & Weir [15]. This model partitions genetic variance into the additive ‘nuclear’ and ‘extranuclear’ effects of the female and male parents and their two-way non-additive interactions (figure 2; electronic supplementary material, table S4). The nuclear effects are the effects of genes transmitted from parents to offspring that are independent of the sex of the parent. The nuclear effects are only manifested after eggs are fertilized and are a property of the progeny genotypes. Extranuclear effects refer to any parent-specific effect that is independent of the nuclear genome passed on to the progeny. Extranuclear effects include maternal or paternal genetic effects; parent-of-origin effects of the nuclear genome, such as epigenetic modifications; and maternal cytoplasmic contributions to eggs. Remarkably, our analysis revealed that 85% of the total genetic variation and 33% of the total phenotypic variation in productivity was due to the extranuclear component of the female parents (figure 2; electronic supplementary material, table S4). By contrast, variation due to the nuclear genomes of the progeny was not significant.

We performed a genome-wide association study (GWAS) to identify common (minor allele frequency or MAF ≥ 0.10) single-nucleotide polymorphisms (SNPs) associated with variation in productivity of female parents. We focused on only the female component of productivity here as it accounted for the vast majority of variation. Thus, the mapped trait is sex-limited and female specific. Among the 1 441 610 SNPs tested, we found a total of 15 with *p*-values < 10^−5^ associated with productivity of females, within or near (within 2 kb of the start and end sites) nine genes (electronic supplementary material, table S5). The most significant SNP (*p* = 3.42 × 10^−7^) is 1612 bp downstream of the gene encoding the *X-*linked *dopamine 2-like receptor* (*Dop2R*) gene (electronic supplementary material, table S5) and explained approximately 47% of the genetic variance of productivity in females. On average, one minor G allele in females increased productivity by 13.92 flies compared to the T allele when crossed with any male genotype (electronic supplementary material, table S5). Female fertility is positively correlated with body size in many insects. Indeed, we found a significant positive correlation between the female component of productivity and female thorax length [16] (*r* = 0.35, *p* = 0.01) but not thorax width (*r* = 0.16, *p* = 0.28). We therefore tested whether the GWAS associations were mediated by the variation in body size by testing for SNP-productivity associations conditional on thorax length. The significance of the tests either remained or diminished very slightly when conditioning the tests on thorax length (electronic supplementary material, table S5), suggesting that the association cannot be explained by the relationship between fertility and body size.

**Figure 3.**
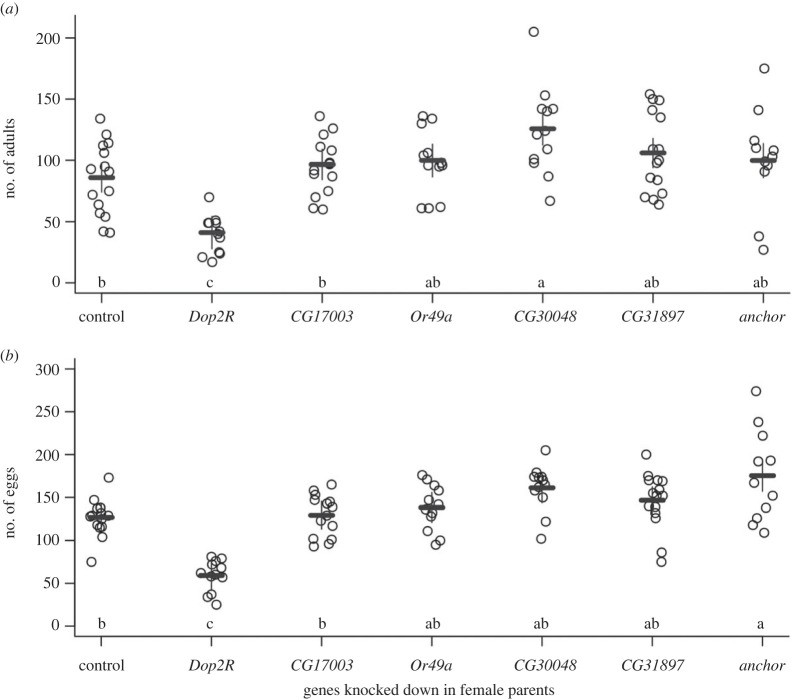
Effects on productivity of RNAi knockdown of candidate genes in female parents. (*a*) The number of adult progeny and (*b*) the number of eggs from *actin-GAL4* > *UAS*-RNAi females for six candidate genes, each crossed to males from representative DGRP lines spanning the range of male genetic variation in productivity. The control is *actin-GAL4* > RNAi progenitor strain females crossed to males of the same three DGRP lines. The horizontal and vertical bars indicate least-squares means and standard errors, respectively. Genes with the same letter do not have significantly different numbers of adults or eggs based on Tukey's multiple comparison at an alpha level of 0.05.

**Figure 4.**
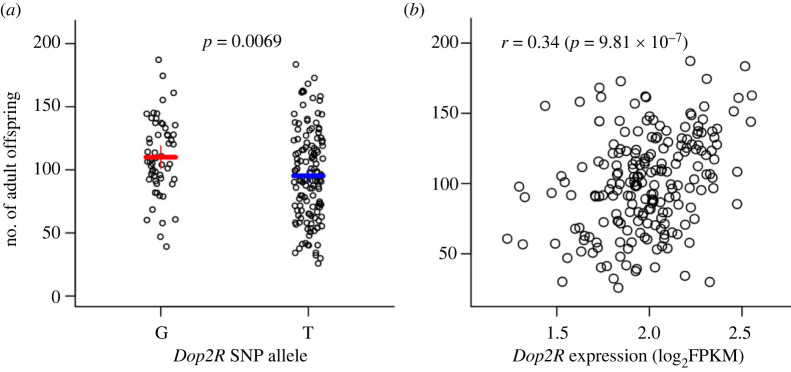
Effects of the *Dop2R* SNP on productivity. (*a*) The number of adult offspring in self-crosses of all DGRP lines according to the *Dop2R* SNP alleles. (*b*) Scatter plot showing the relationship between *Dop2R* expression (log_2_FPKM) and productivity.

Given the lenient significance threshold and small sample size (50 DGRP lines), the GWAS is likely prone to false-positive associations and overestimation of genetic effects. We therefore obtained additional functional and genetic evidence to corroborate the GWAS results. To validate genes implicated by the significant SNPs in GWAS, we performed two additional analyses. First, we used transgenic *UAS*-RNA interference (RNAi) lines driven by an ubiquitously expressed *GAL4* driver (*actin-GAL4*) to knockdown expression of candidate genes in females and crossed them to males of three DGRP lines with high, moderate and low male components of productivity. The RNAi knockdown was performed in females only as we concentrated on the female component of productivity. The reduction in scale achieved by focusing on GWAS hits allowed us to further separate productivity into its two components, egg laying and embryonic survival, both of which could potentially be affected by the female parents through extranuclear effects. We therefore counted the number of eggs laid by females and the number of viable adult flies emerging from the crosses. Among the eight genes with publicly available *UAS*-RNAi lines, two (*l(2)37Bb* and *Rpn3*) did not produce viable flies when knocked down. Of the remaining six genes, only knockdown of *Dop2R* in females differed from all other knockdowns and the control. *Actin-GAL4* > *UAS-Dop2R* RNAi females had significantly reduced productivity compared to the *Actin-GAL4* > RNAi progenitor strain control as well as RNAi of all other genes (figure 3*a*). Furthermore, the effect of *Dop2R* occurred before and/or when eggs were laid because there was also a significant reduction in egg numbers (figure 3*b*). Therefore, we chose to investigate *Dop2R* in more detail, although knockdown of *CG30048* was associated with increased female productivity and knockdown of *anchor* was associated with increased egg laying. In the second analysis, we independently measured the productivity of all (*n* = 204) DGRP females crossed to males of the same lines, including 155 new lines (one line was only present in the diallel experiment). The correlation between the self-crosses in the diallel and this new independent dataset was high (*r* = 0.46, *p* = 8.60 × 10^−4^ for *n* = 49 common lines between the two datasets), testifying to the high heritability and repeatability of the productivity trait. We tested association between the 15 top SNPs from the diallel GWAS and productivity of the self-crosses in the new independent dataset. Among all 15 SNPs tested, three SNPs remained significant (*p* < 0.05) (electronic supplementary material, table S5), including the *Dop2R* SNP. The *Dop2R* SNP had the strongest effect (*p* = 0.0069) (electronic supplementary material, table S5; figure 4*a*) consistent with the diallel experiment.

*Dop2R* encodes a G-protein-coupled receptor for the biogenic amine dopamine (DA) [17] and is an autoreceptor that regulates the release of DA [18]. DA acts as a neurotransmitter, neuromodulator and neurohormone in *D. melanogaster* and other insects. In its role as a neurohormone, DA regulates the levels of juvenile hormone (JH) and 20-hydroxyecdysone (20E), which are required for female oogenesis and fertility; and reduced levels of DA result in abnormal oogenesis and reduced fertility [19–21]. In *D. melanogaster*, pharmacologically decreased levels of DA in sexually mature females (but not males) results in an increase in JH levels [22]. Reduced expression of *Dop2R* in the *corpus allatum* (the endocrine gland in which JH is synthesized) in *D. melanogaster* females using RNAi increases JH levels [23]. Both increases and decreases of JH are associated with defects in oogenesis [22]. Therefore, we hypothesize that the association of *Dop2R* with variation in female productivity in the DGRP may at least partially arise from naturally occurring variation in expression of *Dop2R* in DGRP females, which then causes variation in the amount of DA that regulates fertility via changes in JH and 20E titers. *Dop2R* expression is genetically variable in the DGRP, with a broad sense heritability in females approximately 0.70 [16]. We found a strong correlation between *Dop2R* expression in females with productivity (*r* = 0.34, *p* = 9.81 × 10^−7^; figure 4*b*) but a relatively weak effect of the *Dop2R* SNP on expression (*p* = 0.09). The non-significant effect of the lead SNP on expression could plausibly be due to high linkage disequilibrium with the true causal *Dop2R* SNP. To test whether the effect of the *Dop2R* SNP was mediated by the differential expression of *Dop2R* in lines carrying the alternative alleles, we performed a mediation analysis [24] to partition the effect of the *Dop2R* SNP on female fertility into a direct part and a mediated part due to *Dop2R* expression. The mediation analysis revealed that approximately 19% (*p* = 0.09) of the effect of the *Dop2R* SNP on productivity was mediated by the effect of *Dop2R* expression.

It is intriguing that a SNP strongly associated with fertility remained polymorphic in a natural population, and the allele increasing fertility was less frequent than the allele decreasing fertility. There are several non-mutually exclusive explanations. First, although there is a very large genetic component of fertility in the laboratory, the genetic variation in the wild may be much smaller compared to environmental variation; therefore, the fitness effect is small in nature. Second, this SNP or the causal SNP it tags may be highly pleiotropic, and thus the net fitness effect is small, as would be the case if there is antagonistic pleiotropy with a second fitness trait. Finally, there may be genotype by environment interaction such that the fitness effect varies in fluctuating environments, or at different ages of the flies. This could lead to fluctuation of allele frequency and may allow both alleles to persist.

## 3. Methods

### (a) Fly husbandry and phenotyping

We arbitrarily selected 50 DGRP lines (DGRP_21, DGRP_26, DGRP_28, DGRP_40, DGRP_41, DGRP_45, DGRP_57, DGRP_91, DGRP_129, DGRP_138, DGRP_158, DGRP_161, DGRP_176, DGRP_195, DGRP_208, DGRP_217, DGRP_227, DGRP_228, DGRP_233, DGRP_235, DGRP_239, DGRP_256, DGRP_280, DGRP_313, DGRP_318, DGRP_358, DGRP_365, DGRP_367, DGRP_370, DGRP_375, DGRP_379, DGRP_380, DGRP_386, DGRP_398, DGRP_399, DGRP_461, DGRP_513, DGRP_517, DGRP_555, DGRP_595, DGRP_639, DGRP_705, DGRP_712, DGRP_790, DGRP_805, DGRP_810, DGRP_820, DGRP_882, DGRP_890, DGRP_908) and performed a full diallel cross, including all reciprocal and within-line crosses. To measure productivity, we placed four virgin females and four males from the respective lines in a vial for 2 days before removing the adults. We counted the total number of adult females and males up to 16 days post-mating. We performed three replicates for each of the 2500 possible crosses.

### (b) Quantitative genetic analysis

We performed quantitative genetic analyses using SAS PROC MIXED. To test for the effect of *Wolbachia* infection, we fitted a mixed model with *Wolbachia* infection for females and males and their interaction as fixed effects, and line nested within the *Wolbachia* infection as a random effect. We adjusted all observed phenotypic values by the estimated effects of *Wolbachia* infection for subsequent analyses. To partition phenotypic variance, we first fitted a simple model with cross as a random effect. The cross variance component is thus the genetic variance. To partition phenotypic variance according to the bio-model, we computed covariance matrices using relatedness among the crosses such that the covariance between full sibs is $\sigma_{f}^{2}+\sigma_{m}^{2}+\sigma_{\;fm}^{2}+2\sigma_{n}^{2}+\sigma_{nn}^{2}$, between reciprocal full sibs is $2\sigma_{n}^{2}+\sigma_{nn}^{2}$, between maternal half sibs is $\sigma_{f}^{2}+\sigma_{n}^{2}$, between paternal half sibs is $\sigma_{m}^{2}+\sigma_{n}^{2}$ and between reciprocal half sibs is $\sigma_{n}^{2}$; where $\sigma_{f}^{2}$, $\sigma_{m}^{2}$ and $\sigma_{\;fm}^{2}$ are the variance components for female and male parents’ extranuclear effects and their interaction, respectively; and $\sigma_{n}^{2}$ and $\sigma_{nn}^{2}$ are the variance components for the nuclear genome's effect and interaction between the two paternal and maternal nuclear genomes, respectively [25].

### (c) Single-nucleotide polymorphism calling and genome-wide association study

We used processed alignment files produced by the DGRP Freeze 2 [14] to call SNPs using JGIL [26]. We called SNPs *de novo* to recover sequences for DGRP_398, which was removed from DGRP Freeze 2 because of its relatedness with DGRP_383. We required that SNPs pass a variant level quality score of 500, genotype score of 20, maximum error rate of 0.01 and a minimum allele frequency of 0.1 among the 50 lines. All segregating genotypes within an inbred line were removed from further analysis and thus considered missing. We used the means of productivity across all crosses for the respective female parents as phenotypes for gene mapping, thus mapping only the female component of productivity, which explained the overwhelming majority of variation. None of the major polymorphic inversions or principal components of the genotypes was significantly associated with the trait. We performed simple linear regressions for GWAS using PLINK [27]. To test for effects of body size, we fitted additionally a model where thorax length was included as a continuous covariate. Female gene expression for the *Dop2R* gene was obtained from a previous study in which whole-body expression was quantified by RNA-Seq separately in males and females [16]. Mediation analysis was performed using the mediation package in R [24]. The mediation function in this package takes two models, a mediator model that connects the SNP with gene expression and an outcome model where the phenotype is determined by both the SNP genotype and gene expression, and partitions the effect of the SNP into the average causal mediation effect (ACME) and the average direct effect. The ACME can be considered as a model where the SNP first changes gene expression, which subsequently affects organismal phenotype.

### (d) RNAi and genetic tests for candidate genes

To functionally test candidate genes, we crossed each of the *UAS*-RNAi transgenic lines to the ubiquitous *actin-GAL4* driver line. The *UAS*-RNAi lines were obtained from the Vienna Stock Center, including *CG17003* (*P{KK105603}VIE-260B*), *Dop2R* (*w*^1118^;*P{GD732}v11471*), *Or49a* (*w*^1118^;*P{GD732}v11471*), *CG30048* (*P{KK105490}VIE-260B*), *DptB* (*P{KK112183}VIE-260B*), *Rpn3* (*P{KK101504}VIE-260B*), *l(2)37Bb* (*P{KK104117}VIE-260B*), *anchor* (*P{KK101388}VIE-260B*), *CG31897* (*P{KK106122}VIE-260B*). The adult female offspring (where the respective genes were knocked down) were then crossed to each of three DGRP lines representative of high, medium and low male genetic effects on productivity (DGRP_367, DGRP_375, DGRP_379). The adult flies were transferred 24 h later to a new vial and visible eggs were counted. The flies in the new vial were cleared another 24 h later and eggs were counted again. The total number of eggs and adults that emerged were used for subsequent analyses. Five biological replicates for each of the crosses were performed. To independently test the top 15 SNPs from the productivity diallel GWAS, we measured the productivity of self-matings for all DGRP lines. We crossed four females and four males of each line in each of 10 replicate vials, and measured productivity as described above for the diallel cross analysis.

## Data Availability

The raw data and computer codes used in this study are available in a GitHub repository https://github.com/qgg-lab/dgrp-diallel [28]. The data are provided in the electronic supplementary material [29].
